# Beyond Epistaxis: A Rare Case of Ectopic Sinonasal Adamantinomatous Craniopharyngioma

**DOI:** 10.7759/cureus.68357

**Published:** 2024-09-01

**Authors:** Kabilen Selva Kumaran, Nurul Syamiza Shamsudin, Harvinder Singh Dalip Singh, Philip Rajan Devesahayam

**Affiliations:** 1 Otolaryngology - Head and Neck Surgery, Hospital Raja Permaisuri Bainun, Ipoh, MYS; 2 Otolaryngology - Head and Neck Surgery, Hospital Tuanku Ja'afar, Seremban, MYS

**Keywords:** adamantinomatous craniopharyngioma, ectopic, epistaxis, ethmoid sinus, frontal sinus, sinonasal tumour

## Abstract

Adamantinomatous craniopharyngioma (ACP) is one of the two types of craniopharyngioma recognized by the World Health Organization (WHO), the other being papillary craniopharyngioma (PCP). These rare, benign tumours of the pituitary region are classified as Grade 1 central nervous system (CNS) tumours. ACP predominantly affects adolescents aged 5-15 years and adults over 50 years. It is usually located in the sellar and suprasellar regions. We present the case of an 18-year-old Malaysian female with a six-year history of persistent epistaxis and progressive nasal obstruction, an atypical presentation of ACP. This report highlights an entirely ectopic location of ACP in the sinonasal region. The tumour encompassed the left nasal cavity, the left anterior and posterior ethmoid sinuses, and the bilateral frontal sinuses. The unusual presentation of this tumour was detected with the aid of CT and MRI and confirmed by histopathological examination. In this case report, we discuss a rare presentation, an unusual location, and the strategies employed to overcome these challenges.

## Introduction

Craniopharyngiomas are infrequent, benign tumors originating from the epithelial remnants of the craniopharyngeal duct or Rathke's pouch. Typically located in the sellar or suprasellar regions, their occurrence in other areas is extremely rare. There are two documented variants of the condition: the adamantinomatous craniopharyngioma (ACP), which commonly presents in childhood, and the papillary craniopharyngioma (PCP), which occurs in adulthood [1]. Ectopic craniopharyngiomas are exceptionally rare, with nasal presentations being particularly unusual. This report explores a distinctive case of sinonasal ACP that diverged from typical instances seen in intracranial sites and presented unique diagnostic challenges [2-3].

Craniopharyngiomas typically present with headaches, visual symptoms such as temporal hemianopsia, and hormonal deficiencies, which can manifest as amenorrhoea in females, erectile dysfunction in males, and diabetes insipidus [4]. However, in this case, the patient presented uniquely with recurrent epistaxis and nasal obstruction, attributed to the ectopic location of the craniopharyngioma. Detailed evaluations, including nasal endoscopic biopsy and imaging modalities such as MRI and CT, led to the diagnosis of sinonasal ACP. The rarity of this tumor in the nasal cavity highlights the need to discuss its pathogenesis and the importance of considering ectopic sites in patients with unexplained nasal symptoms.

## Case presentation

**Figure 1:**
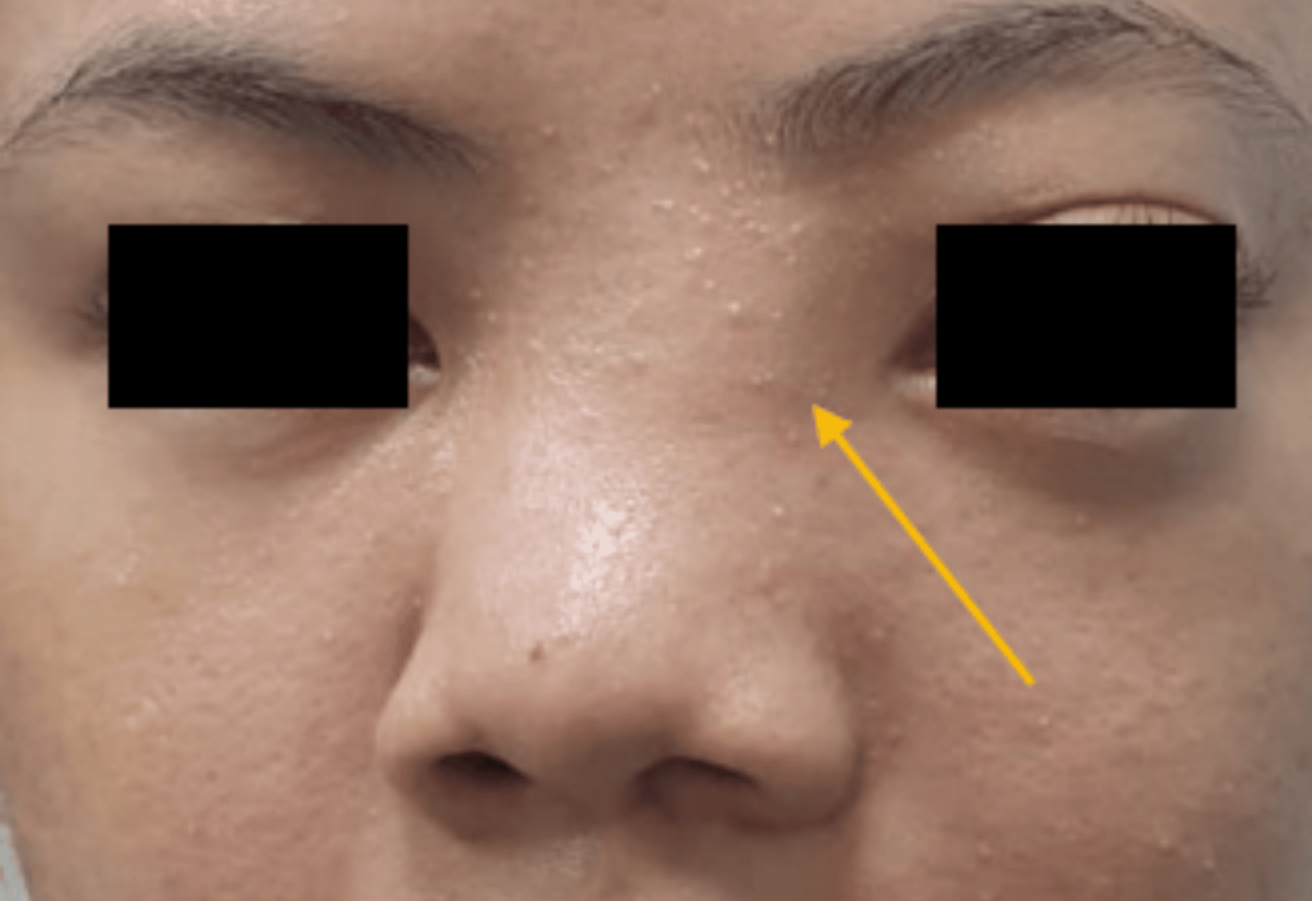
Anterior view of the patient's face Arrow: increased left intercanthal distance with fullness, displacing the nose towards the right

An 18-year-old Malaysian girl with no known medical illnesses was referred to the ENT clinic by a general practitioner due to persistent epistaxis over the past six years. Her epistaxis was spontaneous, intermittent, and minimal, originating from the left nostril. She also complained of left nasal blockage persisting for the same duration, which progressively worsened to involve the right nostril. She also reported anosmia and occasional cacosmia. Furthermore, she exhibited left-sided facial swelling (Figure 1), which had been progressively increasing in size over the past two months, accompanied by blurring of vision primarily on the same side. There were no other intracranial symptoms such as headache, nausea, vomiting, limb weakness, or altered mental status. She also denied any weight loss or loss of appetite and had no family history of malignancy. Her past medical and surgical history was unremarkable.

Anterior rhinoscopy revealed a polypoidal mass with punctate bleed, occupying the entire left nasal cavity until 0.5 cm from the vestibule, pushing the nasal septum to the opposite side. The mass was non-tender, and visualization beyond it was not possible. A cold spatula test revealed absent misting bilaterally. Minimal proptosis was noted in the left eye compared to the right, but there was no ophthalmoplegia or diplopia. The examination of the oral cavity, ears, and neck was unremarkable. Cranial nerves III, IV, V, VI, VII, IX, X, XI, and XII were intact.

**Figure 2:**
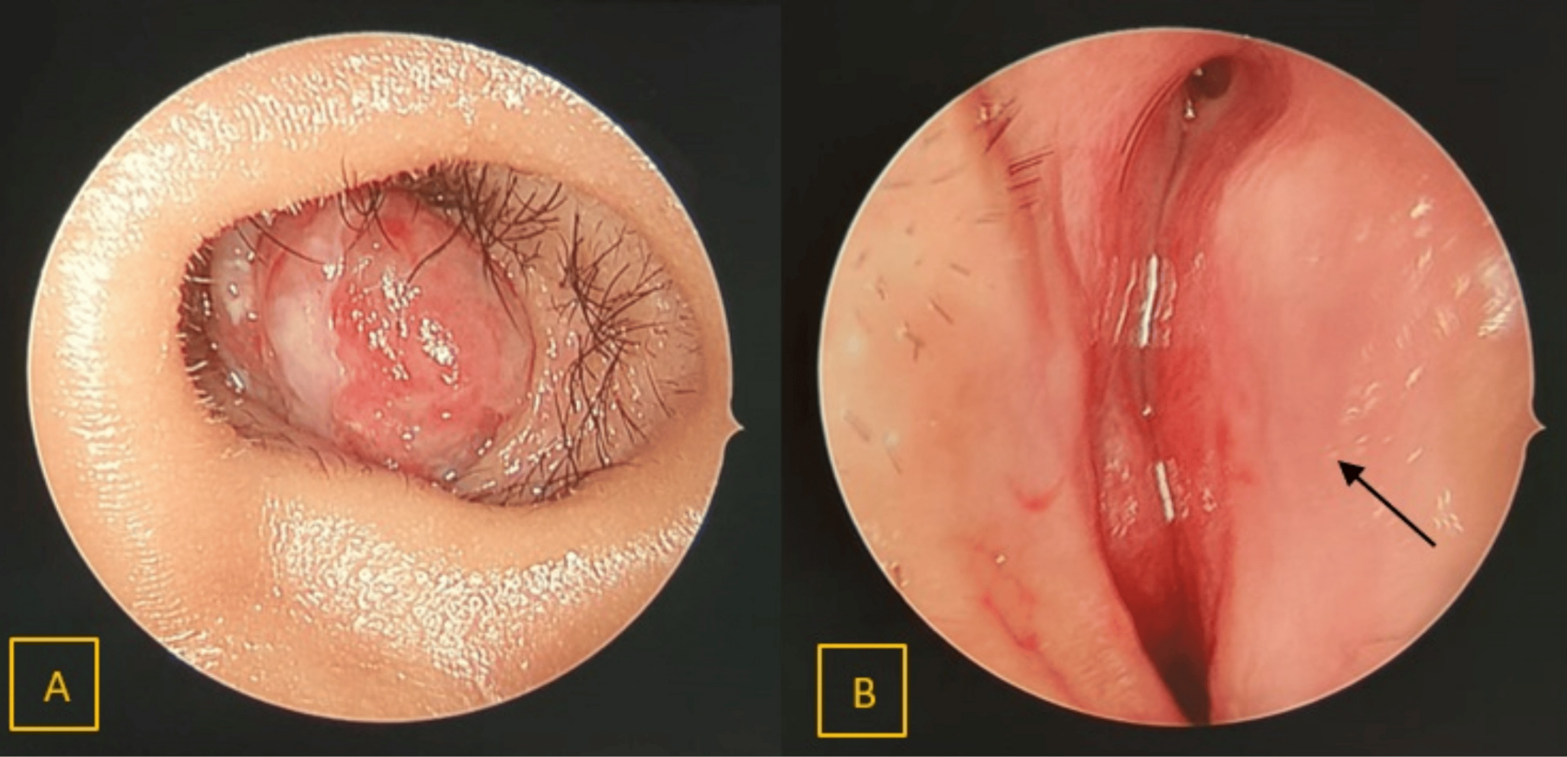
Endoscopic view (A) Left nostril: a polypoidal mass is seen. (B) Right nostril. Arrow: deviated nasal septum to the right occluding the right nasal cavity

Rigid nasoendoscopy revealed the mass completely obstructing the left nostril, rendering it impossible to view beyond the mass. Owing to the mass effect, there was a severe septal deviation to the right, hindering nasoendoscopic passage through the right nostril as well (Figure 2).

**Figure 3:**
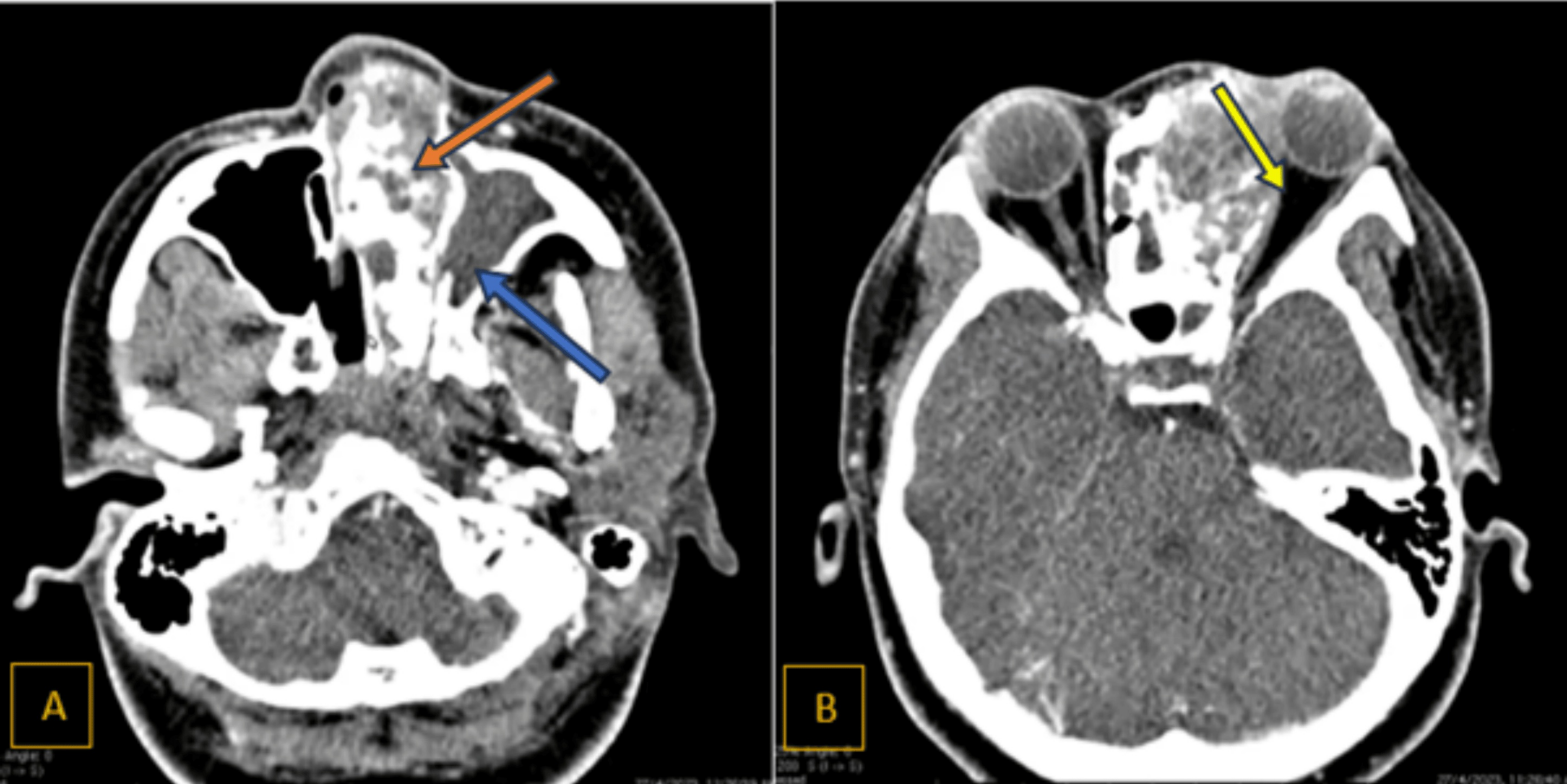
Axial view of CT with contrast (A) Orange arrow: heterogeneous enhancing mass over left nasal space. Blue arrow: erosion of the medial wall of the maxillary sinus with extension into the left maxillary sinus. (B) Yellow arrow: mass abutting the medial rectus muscle on the left side; however, the muscle is preserved CT: computed tomography

**Figure 4:**
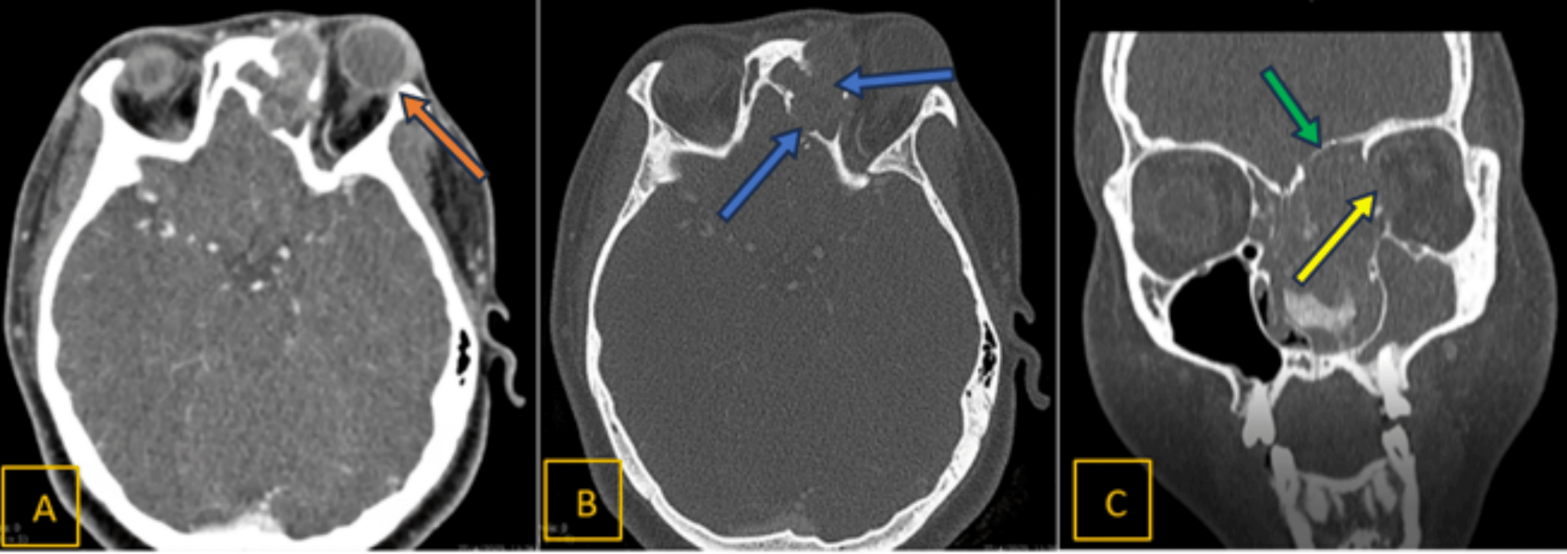
CT with contrast: different views (A, B) Axial view. (C) Coronal view. Orange arrow: mild ptosis of the left eye. Blue arrow: the anterior and posterior walls of the frontal sinus are eroded by the mass. Yellow arrow: eroded lamina papyracea. Green arrow: erosion of the superomedial wall of the left frontal sinus CT: computed tomography

A CT scan of the paranasal sinuses showed a large heterogeneously enhancing mass measuring 7.3 x 3.0 x 5.5 cm occupying the left nasal cavity (Figure 3) and left ethmoid sinus with internal coarse calcification. The mass extended anteriorly to the left vestibule, posteriorly abutting the left nasopharynx, medially compressing the right nasal cavity and laterally compressing the left maxillary medial wall with bony destruction; it was inferiorly limited by the hard palate. The left maxillary sinus was opacified by the obliteration of the left osteomeatal complex. Superiorly, the mass eroded and infiltrated the inferior and superior walls of the frontal sinuses (Figure 4), the left lamina papyracea, and the medial wall of the left orbit, extending into the medial aspect of the left extraconal space and abutting the left medial rectus extraocular muscle and left frontal sinus. The mass abutted the left frontal extradural space of the anterior cranial fossa. These findings suggested a large left sinonasal tumor with local infiltration.

**Figure 5:**
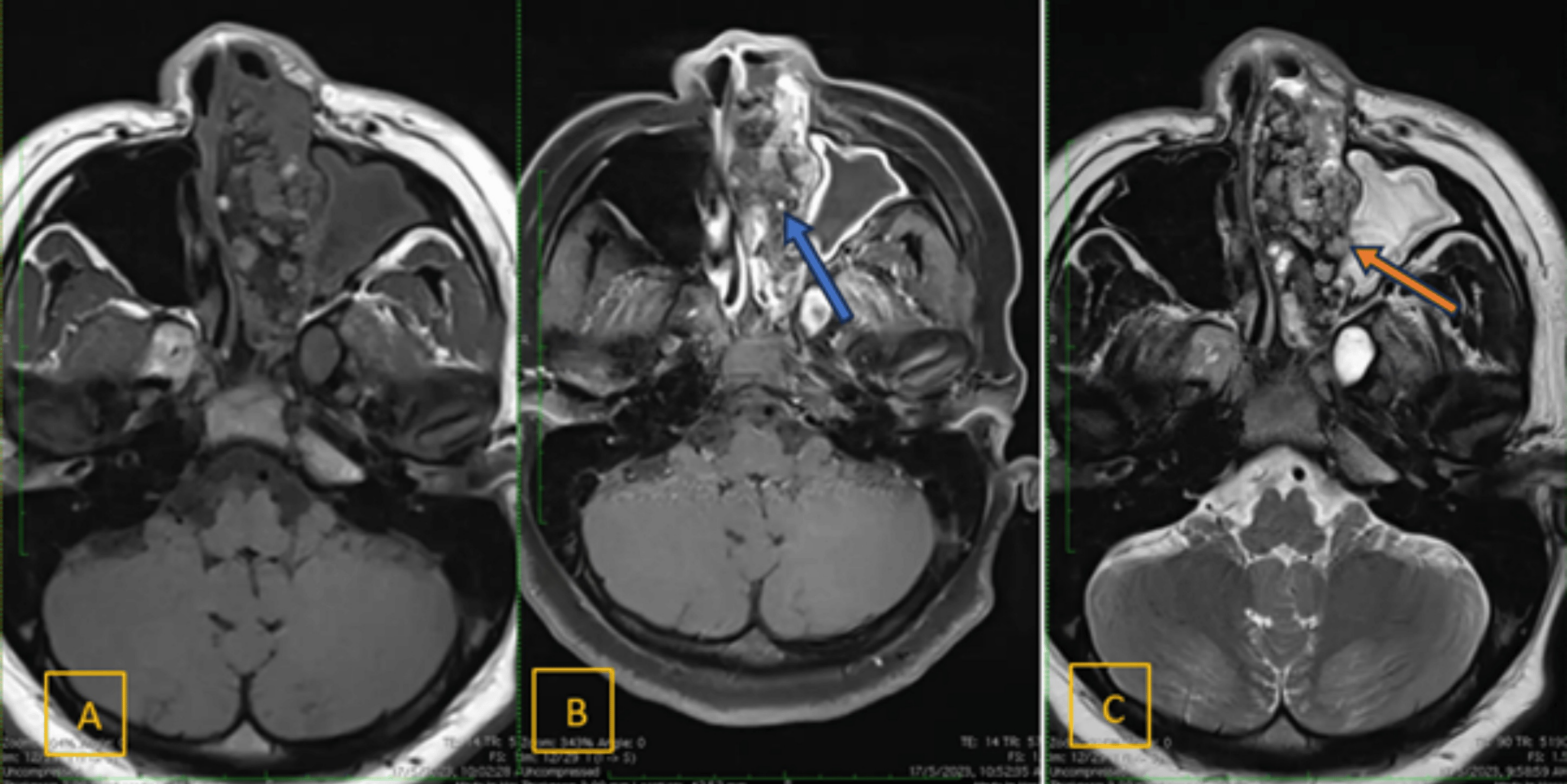
MRI axial view (A) T1-weighted pre-contrast. (B) T1-weighted post-contrast. (C) T2-weighted. Blue arrow: heterogeneous enhancement mass occluding the left nasal passage. Multiple intratumoral calcifications and T1 hyperintense signals due to proteinaceous fluid or hemorrhagic components. Orange arrow: erosion of the left medial wall of the maxillary sinus MRI: magnetic resonance imaging

An MRI of the brain, paranasal sinuses, and orbits revealed a large enhancing lobulated and expansile mass occupying the left nasal cavity, extending to the left ethmoid sinuses and bilateral frontal sinuses, measuring 6.0 x 2.4 x 5.3 cm (Figure 5). The mass was solid-cystic with fluid levels and caused compression of the left retrobulbar space, resulting in proptosis of the left eye. There was a loss of the periorbital hypointense line and fat streakiness in the left orbit, with the left medial rectus muscle appearing smaller than the right. The left optic nerve and superior ophthalmic vein were preserved.

**Figure 6:**
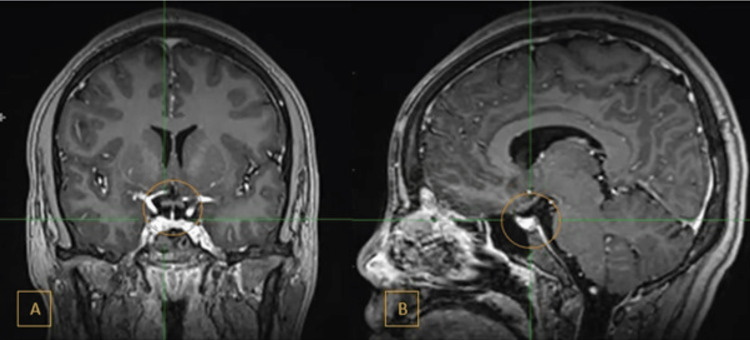
Contrast-enhanced T1-weighted MRI (A) Coronal view. (B) Sagittal view. Orange circle: normal pituitary gland, suprasellar cistern, and optic chiasm MRI: magnetic resonance imaging

The optic chiasm (Figure 6) and visualized optic tracts were also normal. The MRI suggested an aggressive sinonasal tumor with left intraorbital extension.

The patient was also referred to the ophthalmology department due to blurred vision in the left eye. Examination revealed a decrease in visual acuity in her left eye, with a Snellen chart score of 6/12, whereas her right eye maintained a score of 6/6. Otherwise, all extraocular muscle movements and intraocular pressure (IOP) were normal, and there were no signs of nystagmus or diplopia.

**Figure 7:**
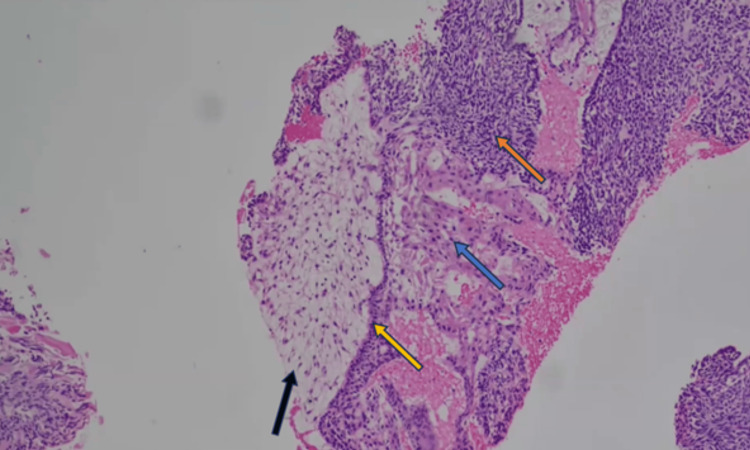
Hematoxylin and eosin microscopic findings at low power magnification Black arrow: cystic degeneration. Orange arrow: stellate reticulum. Yellow arrow: palisading basal cells also known as picket-fence. Blue arrow: squamous epithelium

**Figure 8:**
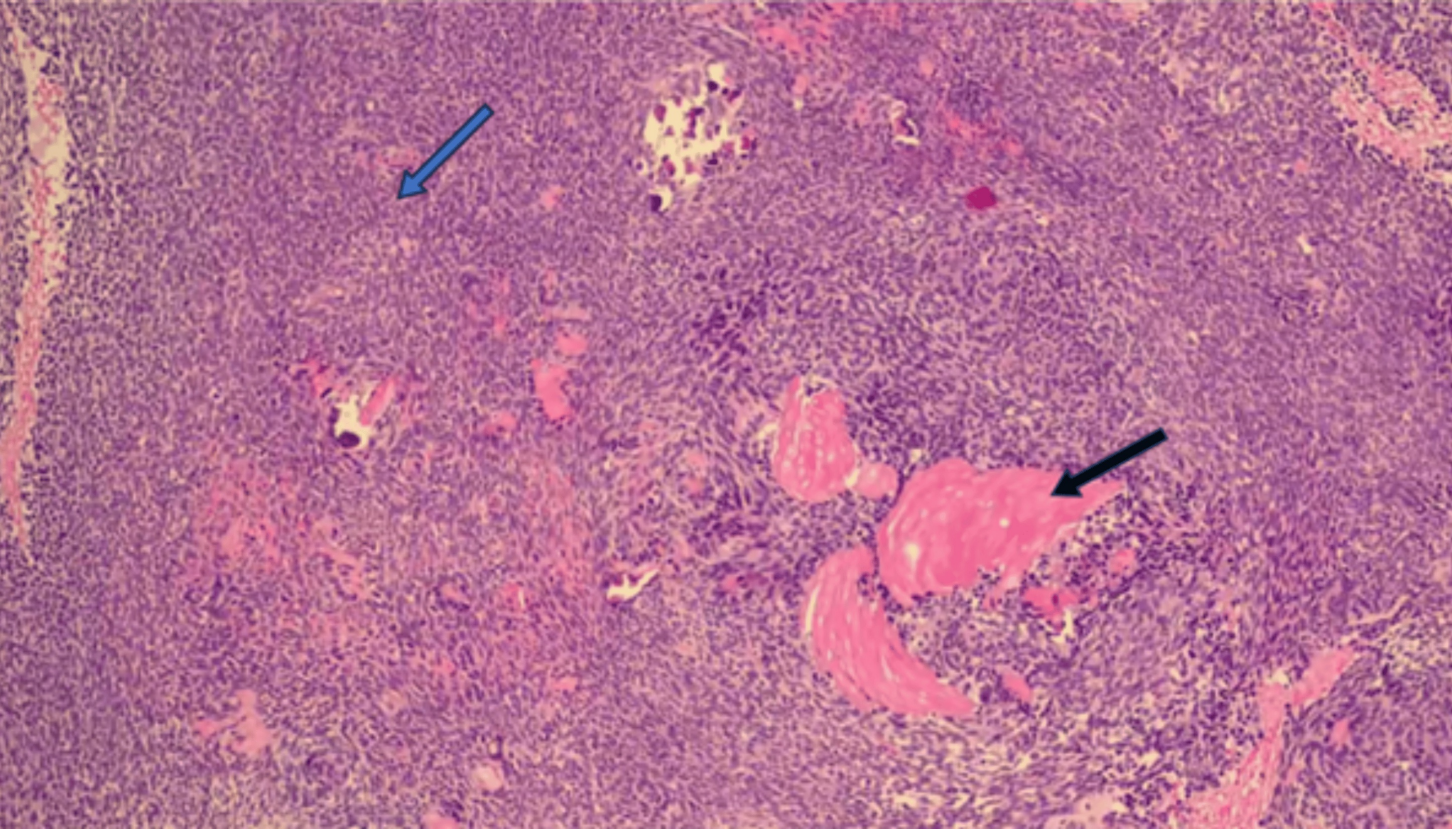
Hematoxylin and eosin microscopic findings at high power magnification Black arrow: wet keratin composed of anucleated squames. Blue arrow: squamous epithelium

**Figure 9:**
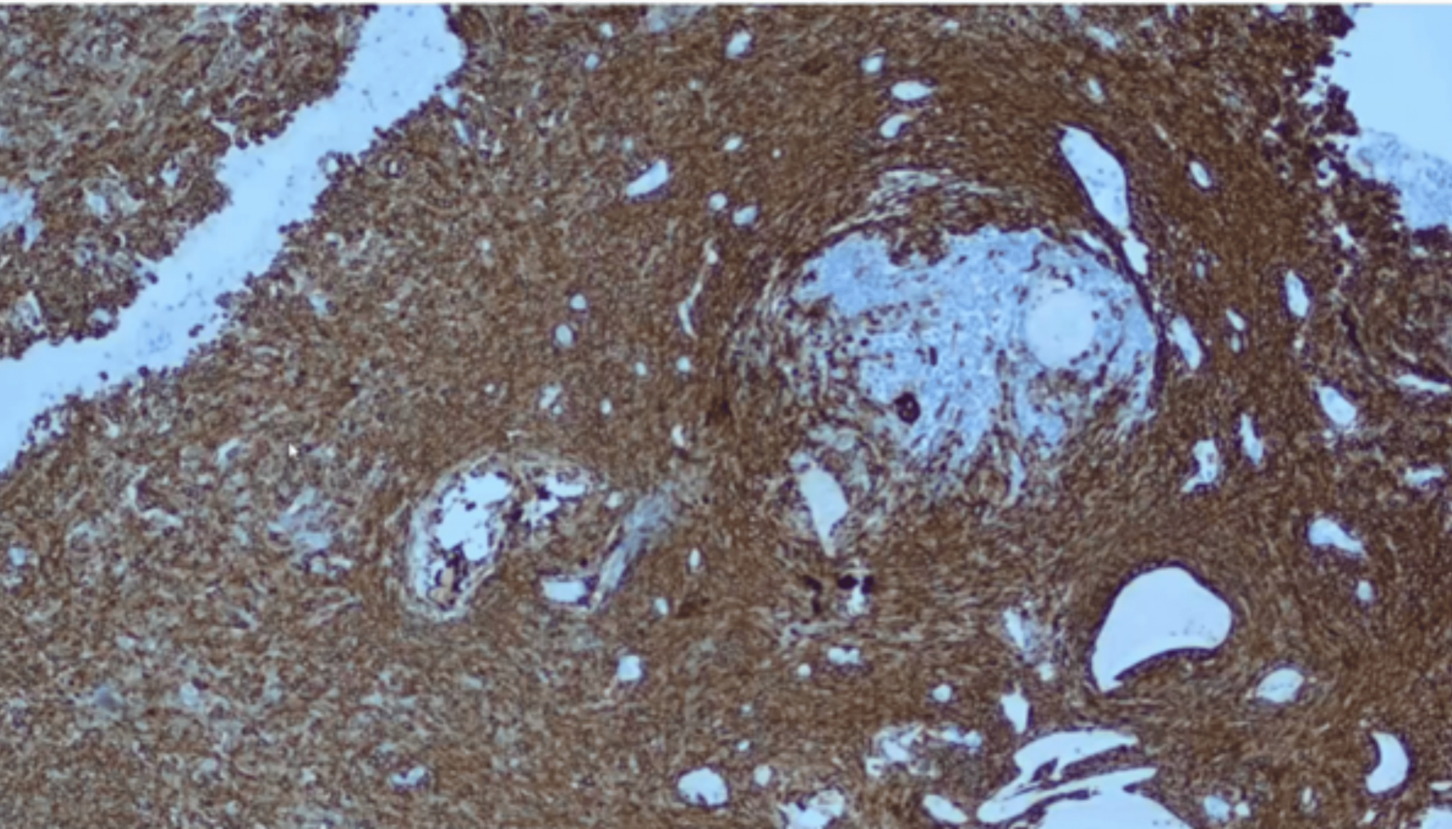
Immunohistochemical CK 5/6 of adamantinomatous craniopharyngioma CK5/6 positivity was noted in the cells, showing intranuclear staining within the squamous whorls

A biopsy of the nasal mass was performed under general anesthesia to ensure better hemostasis. Biopsy showed fragments of tumor tissue composed of neoplastic squamous cells arranged in trabeculae and islands with central areas of stellate reticulum cells (Figure 7). Some tumor islands showed peripheral palisading with the reverse polarity of the basal cells, along with the presence of wet keratin (Figure 8) and calcification embedded in an oedematous stroma. Bony trabeculae with intervening tumor tissue and cystic spaces lined by a flattened epithelium were also observed. The cells were positive for cytokeratin (CK) 5/6 (Figure 9). Malignant cells were not observed in this sample. The histopathological findings were consistent with those of adamantinomatous craniopharyngiomas.

Following a multidisciplinary discussion with the neurosurgical team, endoscopic endonasal tumor removal was performed. Intraoperative findings included a deviated nasal septum, which was pushed to the right by the tumor. The tumor was hard and bony in consistency, extending to the left maxillary sinus and frontal recess, breaching the left lamina papyracea and exposing the periorbital fat. Although the frontal dura mater was exposed, the cribriform plate was not involved, and the dura was not breached. The tumor was then removed in a piecemeal fashion. The exposed dura mater was closed using fibrin sealant, hemostatic agent, and gel foam. The intraoperative specimens confirmed the diagnosis of ACP.

The patient was discharged in good condition and scheduled for a six-monthly follow-up. A postoperative MRI performed six months after the surgery revealed no residual tumor. One year later, the patient showed no signs or symptoms of recurrence, and her vision had improved significantly.

## Discussion

Craniopharyngiomas are rare tumors, accounting for 1-3% of all primary intracranial tumors in adults and 5-10% in children [5]. These slow-growing, non-glial tumors typically occur with a bimodal age distribution, affecting children, adolescents, or adults over the age of 50 years. Although classified by the WHO as benign Grade 1 tumors, they may sometimes be considered 'malignant' due to their potential to disrupt neuroendocrine structures or cause neuropsychological complications [6]. Craniopharyngiomas develop from squamous remnants along the invagination pathway of the primitive stomodeum, known as Rathke's pouch, which extends from the nasopharynx to the hypothalamus [7]. This developmental origin explains why these tumors can occasionally occur at extracranial sites, as outlined in Erdheim's theory [8].

Typically, craniopharyngiomas arise in the suprasellar and sellar regions of the brain, with infrasellar or ectopic extension observed in approximately 5% of the cases [9]. To the best of our knowledge, this is the only documented case of a craniopharyngioma that is purely extracranial and extending from the left nasal cavity to the left anterior and posterior ethmoid sinuses and bilateral frontal sinuses. To fully understand the 'ectopic' nature of our case, it is essential to delve into the embryological development of the pituitary gland.

The pituitary gland develops from two sources: an upgrowth from the ectoderm of the stomodeum and a downgrowth from the neuroectoderm of the diencephalon. During the fourth week of gestation, a diverticulum known as Rathke's pouch forms in the roof of the primitive mouth cavity (stomodeum) and begins to grow towards the brain. By the fifth week, the pouch elongates and narrows at its connection with the oral epithelium, making contact with the infundibulum, a ventral downgrowth from the diencephalon. The stalk of Rathke's pouch extends through the chondrification centers of the presphenoid and basisphenoid bones of the skull. By the eighth week, the connection between the pouch and the oral cavity had disappeared, placing Rathke's pouch in close contact with the infundibulum. However, a remnant of this stalk may persist in the pharyngeal roof, potentially giving rise to a small pharyngeal hypophysis.

Occasionally, the craniopharyngeal duct, which connects Rathke's pouch to the pharynx, does not close completely. This persistent craniopharyngeal duct is found in approximately 0.2% of adults. Anatomically, the craniopharyngeal canal extends from the floor of the sella turcica through the sphenoidal septum into the vomer. As a result, ectopic craniopharyngiomas may develop from the pharyngeal roof, sphenoid body, or floor of the sella [9]. Our case stands out due to the unique location of the craniopharyngioma, which develops away from the typical craniopharyngeal canal. One possible explanation for this unusual occurrence is the presence of mismigrated Rathke’s cleft cells, suggesting that ectopic craniopharyngiomas may arise from epithelial remnants located anywhere along the obliterated craniopharyngeal tract, from the Rathke’s cleft to the floor of the third ventricle [10]. Additionally, ectopic migration of cell remnants from the obliterated craniopharyngeal canal, potentially destined to form tooth primordia, could also account for the atypical location of the tumor. Another hypothesis involves metaplasia of the Schneiderian membrane epithelium, derived from the stomodeal ectoderm, mirroring the developmental processes of the craniopharyngeal canal [11].

Typically, craniopharyngiomas lead to visual problems due to direct compression of the optic pathways. Additionally, they can cause endocrine dysfunction by compressing the hypothalamic-hypophyseal axis, resulting in symptoms such as dwarfism, anorexia, diabetes insipidus, and amenorrhea. Increased intracranial pressure may also occur in the later stages of the disease [12]. However, the presentation in our case was different due to the atypical location, which explains the absence of typical craniopharyngioma features. It is worth noting that epistaxis and nasal blockage are not classically associated with craniopharyngioma [13].

There are two distinct histological variants of craniopharyngioma: the adamantinomatous type, as observed in this case, and the papillary variant, which is more commonly seen in adults. Grossly, the adamantinomatous variant of craniopharyngioma is characterized by the presence of cysts, calcifications, and cholesterol droplets, often resulting in a "motor oil" appearance. The calcified areas contained epithelial tumor cells arranged in distinct patterns, including palisading epithelium, stellate reticulum, and whorl-like structures. The solid components of the tumor consist of wet keratin and calcification foci, whereas the cysts are filled with dark fluid rich in inflammatory mediators and lipids. In contrast, papillary craniopharyngiomas are typically solid rather than cystic and are formed microscopically with well-differentiated, stratified squamous-like epithelium and an anastomosing fibrovascular stroma, resulting in the formation of prominent papillae. The papillary type is well-circumscribed and does not adhere to local structures [7].

CT and MRI scans are essential radiological tools for evaluating craniopharyngiomas, as they provide complementary insights into the tumor's characteristics. CT scans are particularly useful for highlighting bony anatomy and detecting calcifications, which are commonly associated with the adamantinomatous type of craniopharyngioma. In contrast, MRI offers superior visualization of the soft tissue structures of the tumor and its relationship with the surrounding areas. Notably, craniopharyngiomas often present with both cystic and solid components on imaging, and a T1 bright signal intensity on MRI can indicate the presence of high protein content, cholesterol, mild calcification, or hemorrhage, characteristic of the adamantinomatous type [14].

Additionally, calcifications are more commonly observed in the adamantinomatous type than in the papillary type and are more prevalent in pediatric patients. In contrast, adults tend to present with more variability owing to the histological diversity of craniopharyngiomas, which are not uniformly adamantinomatous. Moreover, MRI can aid in distinguishing ACP from PCP in the pituitary region, as ACP often shows encasement of subarachnoid arterial vessels, a feature not typically observed in PCP [15].

Surgery remains the primary treatment for adamantinomatous craniopharyngiomas [13]. The goal is to completely resect the tumor to prevent compressive symptoms and reduce the risk of recurrence. Notably, there have been instances of craniopharyngiomas undergoing malignant transformation [16], underscoring the importance of thorough surgical management. The choice of surgical approach for craniopharyngiomas depends on the tumor location and size. The options include lateral rhinotomy, transsphenoidal, and transcranial approaches.

In our case, endonasal endoscopic surgery was the most logical choice, because the tumor occupied most of the sinuses, excluding the sphenoid sinus. This approach offers the advantage of magnified visualization of the affected areas, using scopes with 0-, 30-, and 70-degree angles to thoroughly examine all sinuses. Additionally, endoscopic surgery minimizes aesthetic disfigurement and reduces patient recovery time. During the procedure, we successfully repaired the defect in the frontal sinus, avoiding the most common complication of this type of surgery, i.e., cerebrospinal fluid (CSF) leakage [5]. Also, since the tumor was not located in the pituitary region, we were able to avoid the internal carotid arteries and optic nerve.

ACPs are invasive, making complete surgical resection challenging in some cases. Therefore, radiotherapy can be used as an adjuvant treatment for residual tumors. Additionally, radiotherapy can be beneficial in cases of disease recurrence, as repetitive surgeries can be debilitating for the patient [5]. The five-year survival rate for craniopharyngioma is 80% overall. However, age significantly impacts this rate: 99% for patients under 20 years old, 79% for those between 20 and 64 years old, and drops to 37% for individuals over 65 years old. The 10-year disease-free survival rate varies between 60 and 96%. This information pertains exclusively to suprasellar craniopharyngiomas, as cases of entirely ectopic craniopharyngiomas are too rare to provide reliable data [2]. Hence, the prognosis of entirely ectopic craniopharyngiomas is not well-established.

We have been following up with the patient for a year, and it is reassuring to report that there have been no recurrences. The patient is currently leading a normal life, free from neurological or endocrine abnormalities, and her initial nasal symptoms of epistaxis and nasal blockage have completely resolved.

## Conclusions

This case report highlights the rare occurrence of sinonasal ACP in the nasal cavity and sinuses, which is an atypical site for this type of tumor. A successful endoscopic endonasal resection was achieved, with no residual tumor on postoperative imaging. This case underscores the importance of considering the rare presentations of craniopharyngiomas in patients with persistent nasal symptoms. This requires comprehensive imaging and a multidisciplinary approach to diagnosing and managing complex sinonasal tumors. We hope this report will contribute to the broader understanding and awareness of ectopic ACP, emphasizing the need for clinical vigilance and thorough diagnostic procedures for atypical tumor presentations.
